# The Relationship Between Psychological Factors and Nutritional Status in Adolescence

**DOI:** 10.3390/children11111365

**Published:** 2024-11-10

**Authors:** Bojana Marinković, Bojan Ćorluka, Mile Vukajlović, Bojan Bjelica, Nikola Aksović, Saša Bubanj, Emilija Petković, Adem Preljević, Ljubiša Lilić, Tatiana Dobrescu, Adina-Camelia Şlicaru

**Affiliations:** 1 Faculty of Philosophy Pale, University of East Sarajevo, 71420 Pale, Bosnia and Herzegovina; bojana.marinkovic@ff.ues.rs.ba (B.M.); bojancorluka@yahoo.com (B.Ć.); mile.vukajlovic@sociolog.rs (M.V.); 2 Faculty of Physical Education and Sport, University of East Sarajevo, 71420 Pale, Bosnia and Herzegovina; vipbjelica@gmail.com; 3 Faculty of Sport and Physical Education, University of Priština–Kosovska Mitrovica, 38218 Leposavić, Serbia; kokir87np@gmail.com (N.A.); ljubisalilic4@gmail.com (L.L.); 4 Faculty of Sport and Physical Education, University of Niš, 18000 Niš, Serbia; sasabubanj@fsfv.ni.ac.rs (S.B.); petkovicemilija@yahoo.com (E.P.); 5 Department of Biomedical Sciences, Sport and Physical Education, University of Novi Pazar, 36300 Novi Pazar, Serbia; apreljevic@np.ac.rs; 6 Department of Physical Education and Sport Performance, Vasile Alecsandri University, 600115 Bacau, Romania; slicaruadinacamelia@ub.ro

**Keywords:** depression, anxiety, stress, eating habits, physical activity, adolescence

## Abstract

Background: The aim of the research is to explore the relationship between psychological factors and nutritional status in adolescence. The issue of adolescent nutrition is related to several psychological problems, as well as to developmental changes during this period. Research on body image dissatisfaction has confirmed that most adolescents are dissatisfied with their bodies. Developmentally oriented scientists are unique in their approach to explaining the problem of body image, focusing on multiple aspects and attempting to understand the intrapersonal and interpersonal factors that are important for the formation of body image. Methods: The sample included 260 adolescents aged 11 to 15. The assessment of sociodemographic factors, emotional states, physical activity levels, and eating habits was included in the questionnaires filled out by the parents (DASS-21 and the Physical Activity & Nutrition Behaviors Monitoring Form questionnaire). Body Mass Index (BMI) was calculated based on information about the height and weight of the adolescents. Results: The results of the correlation between psychological characteristics and BMI indicate a negative low correlation with all psychological aspects: depression (r = −0.25; *p* < 0.05), anxiety (r = −0.30; *p* < 0.05), and stress (r = −0.28; *p* < 0.05). Over 70% of adolescents are classified in the category of normal BMI. Conclusions: The results indicate that subjects who are of normal weight experience less pronounced depression, anxiety, and stress.

## 1. Introduction

Monitoring the weight and height of adolescents is an important process for assessing “normal” growth and development. Since developmental characteristics vary individually, it is common for adolescents of the same age to exhibit different growth rates, particularly in terms of height and body weight. There is a noticeable trend of increasing developmental characteristics. Looking back to the early twentieth century, growth typically continued until around the age of twenty-five. However, today, that phase is generally completed between the ages of eighteen and twenty [1].

Age and the stage of sexual maturity are highly correlated with body weight and body fat percentage [2]. According to the CDC’s sex-specific BMI-for-age growth charts, adolescents are classified as overweight or obese if their body mass index (BMI) is above the 85th or 95th percentile. BMI provides quick, simple, and cost-effective data [3].

For adolescents with a healthy weight, BMI is often calculated preventively to detect early signs of weight gain and take timely action [4]. An increase in height generally leads to a decrease in BMI, while an increase in weight leads to the opposite. The primary factor contributing to childhood obesity is the imbalance between energy intake and energy expenditure [5]. Several authors emphasize that an overweight child is highly likely to become an obese adult [6,7,8,9].

Energy expenditure is closely related to daily activities. The rapid pace of technological development has significantly disrupted the balance between physical activity and leisure time for both adolescents and adults. Increasingly, daily activities are limited to watching television, using mobile devices as the primary “time-killers” for younger generations, and relying on transportation [10]. The lack of physical activity and an inadequate lifestyle directly contribute to obesity in adolescents [11]. Hypokinesia (insufficient movement) triggers mechanisms that can lead to obesity and other health problems [12].

By integrating psychological assessments to explore whether healthy weight is correlated with lower levels of depression, anxiety, and stress, we hypothesized that adolescents with normal weight are less likely to experience depression, anxiety, and stress.

Hence, the aim of the research was to explore the relationship between psychological factors and nutritional status in adolescence.

### 1.1. Research on Social Aspects of Nutrition

When examining the relationship between physical activity and health, it is evident that a low level of physical activity among adolescents and young people directly increases the risk of obesity [13]. Physical activity, by definition, includes any movement performed by activating skeletal muscles, which requires energy consumption, and encompasses both sports and non-sports activities [14]. According to data from the World Health Organization, insufficient physical activity and excess body mass will contribute to 11.5 million new cases of non-communicable diseases by 2050 [15].

The obesity epidemic brings with it numerous health problems, including metabolic disorders, cardiovascular diseases, diabetes, and endocrine issues. Physical activity plays a significant role in preventing obesity by increasing energy expenditure and creating a balance between energy intake and body mass. Research by Curran and associates found that each additional 60 min of sedentary behavior per day is linked to an 11% higher risk of having more than two comorbidities [16].

Previous studies suggest that being overweight can negatively affect emotional well-being in adolescents, leading to low self-esteem and social withdrawal. Factors such as self-esteem and social integration play a key role in understanding this connection. Such studies emphasize the need to promote healthy lifestyle habits, such as proper nutrition and physical activity, as preventive measures for improving mental health in adolescents. Through these safety measures, it is possible to reduce symptoms of depression and enhance the emotional well-being of young people [17,18,19].

To maintain optimal body mass, it is essential to balance energy intake and expenditure. Factors contributing to excess body weight include physical inactivity and changes in dietary habits. According to Clemente-Suárez and associates, modern diets are higher in calories than diets of the past, with more fat and sugar, while complex carbohydrate intake has decreased. Additionally, the consumption of animal products has increased, saturated fat intake is higher, and there is reduced dietary fiber intake due to lower fruit and vegetable consumption [20].

On a global scale, data from 2016 show that (approximately) over 3.19 billion adults were classified as overweight, with more than 650 million being obese, and globally, 41 million children under the age of five were also overweight [21]. Populations from Central Asia, the Middle East, and North Africa, high-income Western countries, Latin America and the Caribbean, Oceania (among women), and Central and Eastern Europe (among men) are most affected, while Southeast Asia has the lowest percentage of obese individuals [22]. Many studies show that obese children are likely to remain obese throughout their lives, leading to social and health problems, such as teasing by peers and the development of depression in later life [23].

Studying the nutritional trends from 2000 to the present, we find that in all parts of the world, the number of obese people has surpassed the number of undernourished individuals [24]. Some theorists equate the sudden rise in obesity with an epidemic or even a pandemic. On a global scale, obesity has become a recognizable issue. Initially, developed countries such as the United States faced this problem, but it has since spread to developing countries as well. According to empirical data, the global obesity rate in 1975 was approximately 3% in men and 6% in women. Today, the largest number of obese individuals (about one-fifth of the world’s population) comes from six highly developed countries: the USA, Australia, Canada, New Zealand, Ireland, and Great Britain. For example, in the United States, more than a third (35%) of the adult population is obese, while more than two-thirds (69%) are overweight [25].

According to research by Pertić and associates, today’s prevalent condition is for a large percentage of children and young people to be physically inactive [26]. Referring to Parizkova’s research, a preschool child typically covers up to 98 km per week. However, with the beginning of formal education, this figure drops to around 8 km per day [27]. As technology develops, especially with the rise of computers and mobile phones, physical activity among children, young people, and even adults has decreased. Young people are increasingly adopting a passive lifestyle, shifting their interests from physical activities to sedentary habits [28].

It is worth noting the research conducted by Kunješić, which examined the relationship between students’ nutritional status and their physical activity. The study found that boys were more physically active than girls in all areas except for housework. Moreover, the more physically active participants had a normal diet, as opposed to the less active, who were more likely to be obese [29]. Participating in various physical activities during childhood enhances motor skills, knowledge, confidence, and motivation, enabling broader participation in physical activities later in life [30].

Melanson argues that the risk of childhood obesity is linked to modern lifestyles, which involve the consumption of fast food, high-calorie drinks, insufficient sleep, lack of physical activity, and prolonged screen time [31]. Drevnovski highlights additional socioeconomic factors that influence children’s nutritional status, including family income, parental education levels, and the urbanization of their place of residence. According to his findings, children from lower socioeconomic backgrounds are more likely to have an inadequate diet, a sedentary lifestyle, and low levels of physical activity, all of which contribute to the risk of obesity [32].

In recent years, obesity among children and adolescents in Serbia, Bosnia and Herzegovina, and most transitioning countries has reached epidemic proportions. Research from 2022 indicates that 15.8% of the surveyed population aged 11, 13, and 15 (N = 3713) is overweight, while three percent of adolescents are obese [33]. An analysis of the 2015 and 2019 Serbian school population health survey shows that overweight and obesity in Serbian children aged 7–9 have increased, unlike in most COSI countries, where rates have remained stable or decreased over ten years [34].

The data obtained following the COSI protocol in Serbia are concerning, given that no positive changes have occurred compared to the period at the beginning of the millennium, when children and young people most often spent their free time engaging in sedentary activities (watching television, doing homework, listening to music, etc.), with only a quarter of the subjects participating in sports. Additionally, a significant percentage of children and youth avoid mandatory physical education classes in school [35]. This global issue has not spared Bosnia and Herzegovina. According to a study on the health status of the adult population in the Federation of Bosnia and Herzegovina, 37.5% of the population is overweight based on body mass index, while 21.2% fall into the obesity category [36].

### 1.2. Research on Psychological Aspects of Nutrition

Current research indicates that being overweight is a significant factor in mental health. Nutrition is associated with negative moods, anxiety, and depression [37]. In addition to these psychological aspects, body image is also an important factor that changes significantly during adolescence. Body image is a representation of physical appearance and bodily experience [38]. Ramos and associates [39] suggest that during adolescence, there is a relatively high prevalence of weight problems and eating disorders, with body image playing an important role in weight control, eating behavior, and mental health.

One study analyzed the influence of BMI, the perception of being overweight, and body image satisfaction on internalizing symptoms related to mental health in 4531 Spanish adolescents aged 13 to 18 years. The results indicated that the emotional component is related to body image, which is the main predictor of internalizing symptoms in adolescents. Interesting findings were presented regarding how behavior during dieting—whether aimed at losing or gaining weight/volume—and physical activity are related to body image perception and satisfaction, as well as to symptoms of internalization [40].

Research involving adolescent girls indicated that those of normal weight were the most dissatisfied with their appearance, while significantly underweight girls were more satisfied and less anxious about their looks. This finding can be explained by the assumption that individuals of normal weight are in conflict with the prevailing culture of thinness and are more sensitive to social pressures and norms related to appearance. As a result, these individuals may feel less accepted and less attractive [38]. Studies have shown that being overweight is associated with reduced self-esteem due to body appearance, and that these adolescents display increased levels of depression, anxiety, eating disorders, and attention deficit problems [41].

In research that examined the relationship between BMI and quality of life in children, Tara and Ric concluded that being overweight is a significant determinant of negative attitudes related to self-esteem [42]. A study that examined elementary school children with different BMI levels found that students with elevated BMI reported a reduced interest in physical activities, as well as negative attitudes toward their physical abilities due to their increased body weight, which they saw as a limiting factor compared to their peers of normal weight. Additionally, children with elevated BMI tended to adopt habits such as watching TV and spending their free time on computers. The results suggest that schools should implement additional interventions to encourage healthier habits and promote positive attitudes toward physical activities [43].

In research involving a sample of elementary school-aged girls, the findings indicated that emotional and behavioral changes were associated with overweight children. Children with higher BMI and those who were obese belonged to a higher-risk group [44]. There is a significant overlap between obesity, depression, and other comorbidities, as well as a shared pathoetiology [45]. Researchers [46] found a significant association between BMI and depression, showing a positive correlation between obesity and depressive symptoms, where obese individuals have a higher probability of developing depressive symptoms compared to individuals of normal body weight. Furthermore, [47] found a positive association between depressive symptoms and patterns of emotional and uncontrolled eating, and a negative association between cognitive restraint from eating and depressive symptoms in well-nourished individuals.

## 2. Materials and Methods

The study was conducted in June 2024 in two elementary schools, “Srbija” and “Pale Elementary School”, located in Pale, Republika Srpska, Bosnia and Herzegovina. All of the students were tested by pre-trained personnel. Height and weight measurements were taken individually to ensure privacy, particularly since obese adolescents may experience anxiety and concern about their peers’ opinions. Height and weight were measured using the SECA 769 scale, which includes a movable stadiometer for measuring height.

### 2.1. Participants

The research sample consisted of 260 early adolescents, mean age 12.72 ± 1.06 years(Mean ± Std.Dev.), comprising 6th, 7th, 8th, and 9th graders from two primary schools in the Municipality of Pale. The gender distribution of the sample was nearly equal, with 50.8% male participants (132 individuals) and 49.2% female participants (128 individuals). The majority of the adolescents in the sample were born in 2011, representing 32.7% of the total. This was followed by adolescents born in 2012 (26.9%) and 2010 (22.7%). The smallest percentage of adolescents were born in 2009, accounting for just 0.8%. Additionally, 1.2% of the sample did not provide their birth year.

The sample selection was random, with no stratified selection of participants performed. The written consent of the parents/participants’ guardians was obtained prior the study.

The researchers obtained permission from the Ethics Commission of the Faculty of Physical Education and Sports, University of East Sarajevo, to conduct the study (number 1083/24), as well as parental consent.

Only participants aged between 11 and 15, who provided signed consent from both themselves and their parents or guardians and who were willing to complete questionnaires assessing sociodemographic factors, emotional states, physical activity levels, and eating habits (including the DASS-21 and Physical Activity & Nutrition Behaviors Monitoring Form), were included in the study.

### 2.2. Instruments

The instruments used in this research included questionnaires designed to assess sociodemographic factors, emotional states, physical activity levels, and eating habits. Regarding the assessment of socioeconomic status, participants provided responses to questions about the number of members in their households, household income, and household expenses. This information is essential to understanding the socioeconomic circumstances that may affect adolescents’ well-being.

To evaluate depression, anxiety, and stress, the Depression, Anxiety, and Stress Scales by [48] and adapted by [49] were used.

For assessing physical activity and nutritional habits, the standardized and non-modified Physical Activity & Nutrition Behaviors Monitoring Form [50] was utilized (available at [51]), after being translated for research purposes. The nutritional status of each participant was determined based on BMI percentiles. The paper includes BMI categories classified according to percentiles on the CDC growth charts, defined as follows: 1. Below the 5th percentile—underweight; 2. 5th to 85th percentile—normal, healthy weight; 3- 85th to 95th percentile—overweight; 4. Above the 95th percentile—obesity [3].

Body Fat Percentage (BF%) and Basal Metabolic Rate (BMR) were calculated using the following formulas [3]:

BF% = 1.51 × BMI − 0.7 × Age − 3.6 × Gender + 1.4

BMR (for males) = 66 + (13.7 × weight (kg)) + (5 × height (cm)) − (6.8 × age (years))

BMR (for females) = 655 + (9.6 × weight (kg)) + (1.8 × height (cm)) − (4.7 × age (years))

The evaluation team consisted of trained professionals. The data collectors agreed on standard procedures for presenting information to children and their parents, in collaboration with the school counselor. This approach was crucial, given the seriousness of the topic, and allowed for effective communication tailored to the students’ ages. Additionally, standardized methods were used to minimize variations in the interpretation of questions and increase the reliability of the results. These measures ensured not only the validity of the collected data but also adherence to ethical research standards. The participants were evaluated in two sessions, each lasting 45 min, with questionnaires completed by the parents.

Regarding the assessment of height and weight, we emphasize that parents were responsible for reporting these data for their children. To ensure the accuracy and validity of the results, we provided accompanying material in the form of a “training letter.” This material contained clear instructions on properly measuring height and weight, along with recommendations for using standardized measurement methods. This approach allowed parents to provide the most accurate data possible, thereby enhancing the reliability of our research.

### 2.3. Statistical Analysis

SPSS software (SPSS version 24.0, SPSS Inc., Chicago, IL, USA) was used for statistical analysis. Initially, the samples were checked for normality of distribution using the Kolmogorov–Smirnov test. All samples followed a normal distribution (*p* > 0.05). In the next stage, descriptive statistics were determined for each measured indicator. Lastly, the correlation between the test indicators was calculated using Pearson’s correlation method.

## 3. Results

Our results (see Appendix A for a tabular presentation) indicate that the majority of households consist of four members, representing 44.2% of the sample. This is followed by households with five members, which make up 28.8%, and households with three members, accounting for 12.7%. Households with six or more members represent 10.8% of the sample, while the smallest percentage of households have only two members, making up 3.1%. Additionally, 0.4% of the data are missing.

The data show that the majority of households (28.8%) have incomes between 800 and 1200 KM (KM—Convertible Mark; 1.00 Convertible Mark = 0.51 EUR). Additionally, 26.5% of households report incomes ranging from 1200 to 2000 KM, and 18.8% have incomes between 500 and 800 KM. A smaller percentage of households (12.3%) have incomes between 2000 and 3000 KM, while 8.1% fall within the income range of 300 to 500 KM. Only 1.9% of households have incomes above 3000 KM, and 1.2% report earning less than 300 KM. Data on income were missing for 2.3% of households.

The data reveal that the largest percentage of households (38.1%) have monthly expenses ranging from 500 to 800 KM. This is followed by 22.3% of households with expenditures between 300 and 500 KM, and 19.2% with expenses in the range of 800 to 1500 KM. A smaller portion of households (11.9%) report expenditures between 100 and 300 KM, while only 6.9% of households have expenses exceeding 1500 KM. Data on expenditures are missing for 1.5% of households.

These data indicate that relatives play a significant role in food supply for the surveyed households. Specifically, 38.5% of the subjects indicated that less than a third of their total food supply comes from these sources. Additionally, 30.8% of the subjects reported that food from relatives constitutes between a third and a half of their total food supply. Meanwhile, 14.6% of the subjects do not rely on food from relatives at all, and 14.2% stated that more than half of their total food supply comes from this source. Data are missing for 1.9% of households.

In relation to the BMI distribution of adolescents, the majority of the sample, 50.38%, falls within the normal weight category. This is followed by the 43.58% of adolescents categorized as malnourished. A smaller percentage, 4.62%, is classified as above average weight, and only 1.15% of the sample is classified as obese.

Adolescents born in 2012 have a BF% of 13.69%, which is slightly lower than those born in 2011 and 2010, whose BF% are 14.48% and 14.79%, respectively, while interest-ingly, adolescents born in 2009 show a lower BF% of 11.41%.

The BMR values increase steadily from 1336.2 kcal/day for those born in 2012 to 1563.93 kcal/day for those born in 2010.

**Figure 1:**
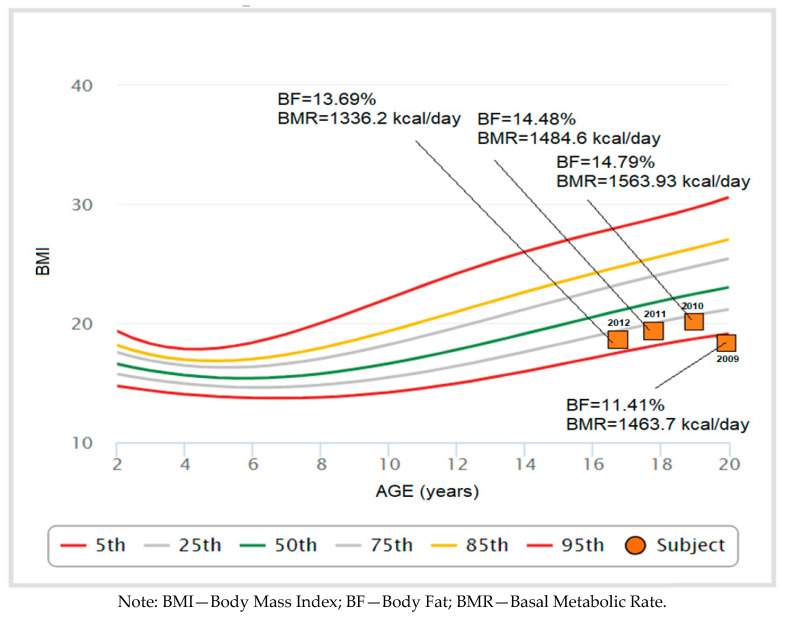
Descriptive indicators for the nutritional status.

However, for those born in 2009, BMR slightly decreases to 1463.7 kcal/day, despite a lower BF% (Figure 1).

Further data indicate that the majority of adolescents (61.92%) are reported to be physically active at an average level, similar to most of their peers, according to their parents’ assessments. A significant portion (18.85%) of adolescents is perceived as much more physically active than most. A smaller group (10.38%) is slightly more physically active than the majority. On the lower end, 3.85% of adolescents are a little less physically active than most, while only 1.15% are reported as being much less physically active. Additionally, 1.54% of parents were unsure about their children’s level of physical activity.

Data on the weekly exercise habits of adolescents, as reported by their parents, reveal that the highest percentage of adolescents exercise three days per week (23.46%), followed by those who exercise five days (18.08%) and four days (16.92%). A smaller percentage exercise two days per week (13.08%). A few adolescents exercise six days (6.15%) or every day of the week (6.54%). Notably, 4.23% of adolescents do not exercise at all, while 7.31% of parents are unsure about their children’s exercise habits. This suggests that a majority of adolescents in this sample are frequently physically active.

The data present the frequency of weekly fast food consumption among adolescents, based on reports from their parents. The highest percentage of adolescents consume fast food once a week (28.46%), followed closely by those who consume it less than once a week (26.92%). A notable portion of adolescents do not eat fast food at all (13.08%). Additionally, 16.54% consume fast food twice a week, while 9.23% report eating it three to five times a week. Only 1.15% of adolescents eat fast food four or more times a week, and 4.23% of parents are unsure about their children’s fast food consumption.

The data show that the largest percentage of the subjects (40%) watch television for one hour or less on weekdays. A notable portion of the subjects (34.2%) watch for two hours, while 10% watch for three hours. Only 4.6% of the subjects reported watching television for four hours, and the smallest group, 2.3%, watches for five hours. Additionally, 3.5% of the subjects do not watch television at all, and 5% were unsure of their television-watching habits. There was a minimal percentage (0.4%) of missing responses.

The data indicate that the majority of the subjects (36.2%) watch television for two hours on weekends, which is higher than on weekdays. Additionally, 22.7% watch for one hour or less, while 14.6% watch for three hours. About 10% of the subjects reported watching television for four hours on weekends, and only 3.5% said they watch for five hours. The smallest group, 1.9%, watches television for six hours or more. A small percentage, 4.2%, do not watch television at all, while 6.5% were unsure of their television viewing habits on weekends. There is a minimal percentage (0.4%) of missing responses.

The data show that the majority of the subjects (62.3%) do not consume carbonated beverages at all. Additionally, 23.8% of the subjects reported consuming carbonated beverages once a day, while 4.2% consume them twice a day. A small percentage of the subjects, 1.2%, consume carbonated beverages three or more times a day. Furthermore, 8.1% of the subjects indicated that they were unsure of their consumption habits, and 0.4% of responses were missing.

The data indicate a notable difference in the consumption of sweetened beverages compared to carbonated beverages. A significant portion of the subjects, 42.7%, consume sweetened beverages once a day, while 23.1% report consuming them twice a day. Additionally, 13.5% of the subjects consume sweetened beverages three or more times a day. On the other hand, 11.9% of the subjects stated that they do not consume sweetened beverages at all. Furthermore, 8.5% of the subjects were unsure of their consumption habits, and 0.4% of responses were missing.

The data show that 35% of the subjects consume chips once a day, which is nearly proportional to the 37.3% of the subjects who do not consume chips at all. A smaller percentage, 9.2%, consume chips twice a day, while only 5% consume them three or more times a day. Additionally, 12.3% of the subjects were unsure about their consumption habits, and 1.2% of responses were missing.

The majority of the subjects (41.5%) consume one glass of milk per day, while 23.5% of the subjects drink two glasses daily. A smaller percentage, 14.2%, consume less than one glass of milk per day, and 8.5% reported drinking three glasses. Only 4.6% consume four or more glasses of milk, while 3.5% do not drink milk at all. Additionally, 3.8% of the subjects were unsure about their milk consumption, and 0.4% of responses were missing.

The data indicate that 36.9% of the subjects consume fruit once a day, followed by 28.5% who eat fruit twice a day, and 28.1% who consume fruit three or more times a day. A small percentage of the subjects, 3.1%, reported not consuming fruit at all, while another 3.1% were unsure about their fruit consumption. Additionally, 0.4% of the responses were missing.

The table reveals that the majority of the subjects (54.2%) consume vegetables once a day, followed by 25.4% who consume vegetables twice a day, and 14.2% who eat them three or more times a day. A small percentage, 2.7%, reported not consuming vegetables at all, while 3.1% were unsure about their vegetable consumption. Additionally, 0.4% of the responses were missing.

### The Correlation Between Psychological Aspects and the Nutritional Level in Adolescence

The psychological aspects examined in this research include depression, anxiety, and stress. Previous studies have indicated that these psychological characteristics are more pronounced in individuals with poor eating habits.

Adolescence is a period during which egocentrism reappears, a psychological concept introduced by [52].

During this stage, teenagers tend to be self-centered, believing that their thoughts, feelings, experiences, and concerns are unique and misunderstood by others.

This is closely linked to body image, which undergoes changes due to cognitive development. Teenagers begin paying more attention to their appearance and how they are perceived by their peers, which becomes important to their self-esteem.

**Figure 2:**
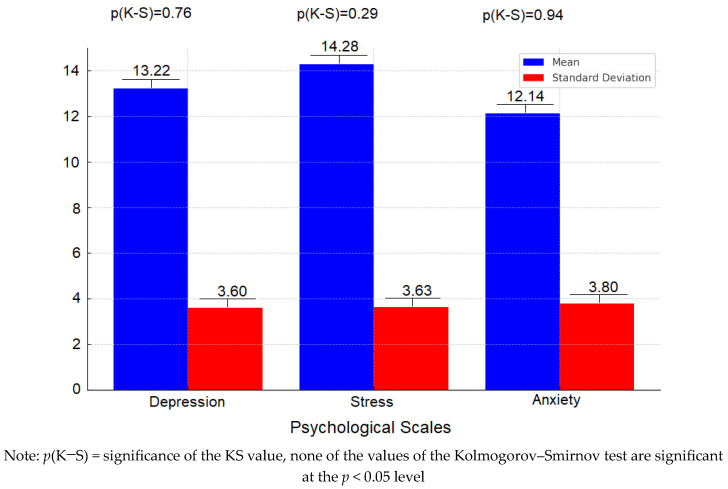
Descriptive indicators for the psychological scales.

Figure 2 presents the descriptive indicators of the psychological scales, as well as the indicators that reflect the normality of the distributions and the reliability of the scales.

Overall, none of the scales show significant deviations from a normal distribution based on the Kolmogorov–Smirnov test, and the reliability values for all scales are acceptable, with α values representing the reliability of the scales, ranging from 0.66 to 0.76 (0.66, 0.68, 0.76, respectively).

**Figure 3:**
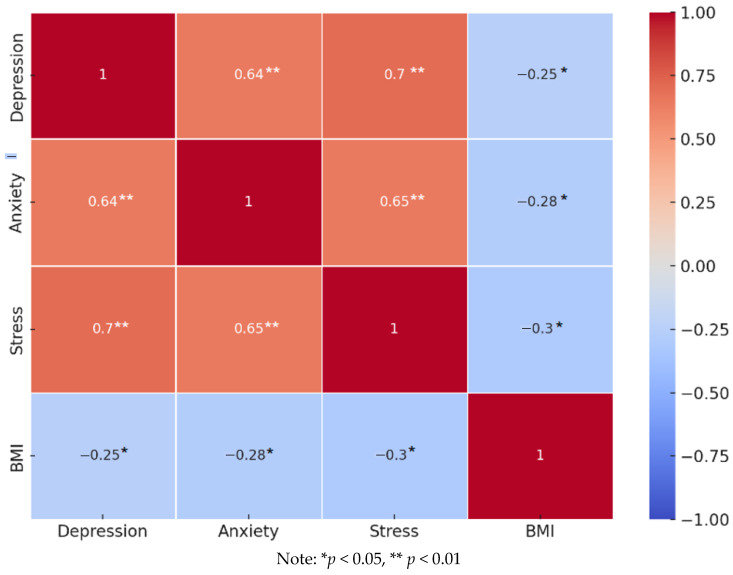
Pearson’s correlation of psychological characteristics and BMI.

The results of the correlation between psychological characteristics, specifically depression, anxiety, and stress, and BMI, are presented in Figure 3.

Based on these results, it can be noted that the psychological variables of depression, anxiety, and stress are negatively related to BMI, with a statistical significance at the 0.05 level. Additionally, the scales for depression, anxiety, and stress are positively correlated with each other at a high intensity.

The findings indicate that the subjects of normal weight exhibit less pronounced levels of depression, anxiety, and stress. Over 70% of adolescents in this sample are classified in the category of normal BMI.

## 4. Discussion

In this study, the research focus is directed toward the relationship between BMI and specific psychological factors, including depression, anxiety, and stress, with BMI used as an indicator of adolescent body mass. The term “nutritional status” is used here in a broader conceptual sense, referring to the general body mass status of participants, but it does not imply a detailed analysis of nutritional intake or dietary status as specific parameters. In this way, BMI serves as a basis for understanding the relationship between body mass and psychological variables, while nutritional status provides the broader framework within which this relationship is explored. The study utilized the “Physical Activity & Nutrition Behaviors Monitoring Form”, completed by the adolescents’ parents, to gather information on their physical activity and dietary habits. This questionnaire provides additional data that support the analysis of adolescents’ nutritional status, offering a broader insight into the context of body mass and psychological factors. Thus, although the study focuses on the relationship between BMI and psychological aspects, the obtained data on nutrition further support the context of nutritional status in which these relationships are examined.

We determined that our participants exercise three days a week, watch television for one hour or less on weekdays, and for two hours per day on weekends. Most of the subjects do not consume carbonated beverages at all, while they tend to consume sweetened beverages once a day. The majority do not eat chips during the day and typically consume one glass of milk per day. Additionally, the largest portion of the subjects eat fruits and vegetables once a day.

In this sample, over 70% of adolescents were of normal weight, and the results support the hypothesis that adolescents with normal weight are less likely to experience depression, stress, and anxiety.

A lower body fat percentage can have a positive impact on adolescents’ mental health, including a reduction in the symptoms of depression. Research has shown that children with lower body fat percentages often exhibit better self-esteem and a more positive body image. This information is directed toward improved social interactions and reduced peer stigmatization, which further decreases the risk of depression. A lower body fat percentage may be associated with higher levels of physical activity, which positively affects mood and mental health. For example, physical activity can increase the production of endorphins, known as “happiness hormones”, which can further alleviate symptoms of depression [53]. Certain groups of authors indicate that there is a significant correlation between body fat percentage and depressive symptoms in adolescents, where lower body fat percentages were associated with lower levels of depression [54]. Additionally, maintaining a healthy body weight and body fat percentage can play a crucial role in preventing mental health issues in adolescents.

However, some research focused on adolescent girls has shown that those who are of normal weight tend to be more dissatisfied with their appearance, whereas significantly malnourished girls report being more satisfied and less anxious, which aligns with the standards of modern society [39].

Our findings suggest that adolescents exercise regularly and that both they and their parents are mindful of maintaining a healthy diet.

As noted by [44], children with higher BMIs and malnutrition belong to a higher-risk group.

Previous research highlights a strong overlap between obesity and depression [45,46,47], which may have influenced parents to encourage their children to participate in physical activities and adopt healthier eating habits.

The data indicate that the adolescents involved in our study have normal body weight, likely due to their habits around physical activity and nutrition, as stated by [55].

The majority of adolescents reported consuming fast food only once a week or less frequently, and a notable percentage do not consume fast food at all.

Most of the adolescents are more physically active than average, so the BMI data obtained align with expectations.

Our findings are practically significant, as they suggest that promoting healthy habits around physical activity and nutrition can contribute to normal weight and psychophysical health.

Based on these results, educational programs for both parents and adolescents could be organized to emphasize the importance of physical activity, healthy eating habits, and the psychological issues of depression, stress, and anxiety related to these factors.

The potential weakness of the study is the is the sample size and its representation. The research was conducted in only two schools from a single municipality, which limits the generalizability of the results to broader populations. The relatively narrow age range (primarily adolescents born between 2005 and 2007) may also limit the ability to assess differences across other age groups in adolescence. Additionally, the sample may not adequately account for diverse sociodemographic backgrounds, which could influence both psychological factors and nutritional behaviors.

A recommendation for future research is to include a more heterogeneous sample, as well as a sample of adolescents receiving treatment for nutritional issues.

The global strategy for improving health through nutrition and physical activity highlights that proper nutrition combined with physical activity represents the best defense against health issues, as mandated by [56,57,58].

Finally, we understand that socioeconomic factors, age, and gender are significant variables that can impact results. Future research should include these variables, as we believe this will enrich the analysis and contribute to developing practical recommendations. This way, the focus will be more directed toward improving the physical and emotional health of adolescents. Such an approach would help explain the complex interactions that affect adolescent health.

## 5. Conclusions

This study examined the relationships between depression, anxiety, stress, and BMI in adolescence.

BMI is determined to be statistically significant in relation to psychological characteristics relevant to mental health.

Adolescents who engage in physical activity tend to develop healthier habits.

The findings emphasize the importance of regular physical activity, balanced nutrition, and parental awareness in promoting both the mental and physical health of adolescents.

## Data Availability

The raw data supporting the conclusions of this article will be made available by the authors on request.

## Appendix A

**Table A1:** Number of household members.

Members	n	(%)
Two	8	3.1
Three	33	12.7
Four	115	44.2
Fri	75	28.8
Six and more	28	10.8
Missing	1	0.4

**Table A2:** Household incomes.

Income	n	(%)
Under 300 KM	3	1.2
From 300 to 500 KM	21	8.1
From 500 to 800 KM	49	18.8
From 800 to 1200 KM	75	28.8
From 1200 to 2000 KM	69	26.5
From 2000 to 3000 KM	32	12.3
Over 3000 KM	5	1.9
Missing	6	2.3

Note: KM—Convertible Mark (1.00 Convertible Mark = 0.51 EUR).

**Table A3:** Household expenditures.

Expenditure	n	(%)
From 100 to 300 KM	31	11.9
From 300 to 500 KM	58	22.3
From 500 to 800 KM	99	38.1
From 800 to 1500 KM	50	19.2
Over 1500 KM	18	6.9
Missing	4	1.5

Note: KM—Convertible Mark (1.00 Convertible Mark = 0.51 EUR).

**Table A4:** Support of relatives in providing food.

Support from Relatives	n	(%)
We do not use that type of food in our diet	38	14.6
Less than a third of the total food is from these sources	100	38.5
Between a third and a half of the total food items	80	30.8
More than half of the total food items	37	14.2
Missing	5	1.9

**Table A5:** BMI of preadolescents.

BMI	n	(%)
Malnourished	114	43.58
Normal weight	131	50.38
Above average	12	4.62
Obese	3	1.15

**Table A6:** Level of physical activity of preadolescents.

Level of Physical Activity	n	(%)
Much more physically active than most	49	18.85
A little more physically active than most	27	10.38
Average, like most	161	61.92
A little less physically active than most	10	3.85
Much less physically active than most	3	1.15
Don’t know	4	1.54

**Table A7:** Days that adolescents spend exercising.

Days of Practice	n	(%)
One day	6	2.31
Two days	34	13.08
Three days	61	23.46
Four days	44	16.92
Five days	47	18.08
Six days	16	6.15
Seven days	17	6.54
Not even a day	11	4.23
I don’t know/I’m not sure	19	7.31

**Table A8:** Frequency of weekly consumption of fast food.

Fast Food Weekly	n	(%)
Less than once a week	70	26.92
Once a week	74	28.46
Twice a week	43	16.54
Three to five times a week	24	9.23
Four or more times	3	1.15
Doesn’t eat this type of food	34	13.08
I don’t know/I’m not sure	11	4.23

**Table A9:** Watching television on weekdays.

Duration	n	(%)
One hour on less	104	40
Two hours	89	34.2
Three hours	26	10
Four hours	12	4.6
Five hours	6	2.3
Not at all	9	3.5
I don’t know/I’m not sure	13	5
Missing	1	0.4

**Table A10:** Watching television on weekends.

Duration	n	(%)
One hour or less	59	22.7
Two hours	94	36.2
Three hours	38	14.6
Four hours	26	10
Five hours	9	3.5
Six hours and more	5	1.9
Not at all	11	4.2
I don’t know/I’m not sure	17	6.5
Missing	1	0.4

**Table A11:** Consumption of carbonated beverages.

Consumption	n	(%)
Once	62	23.8
Two times	11	4.2
Three or more times	3	1.2
Not at all	162	62.3
I don’t know/I’m not sure	21	8.1
Missing	1	0.4

**Table A12:** Consumption of sweetened beverages.

Consumption	n	(%)
Once	111	42.7
Two times	60	23.1
Three or more times	35	13.5
Not at all	31	11.9
I don’t know/I’m not sure	22	8.5
Missing	1	0.4

**Table A13:** Consumption of chips.

Consumption	n	(%)
Once	91	35
Two times	24	9.2
Three or more times	13	5
Not at all	97	37.3
I don’t know/I’m not sure	32	12.3
Missing	3	1.2

**Table A14:** Milk consumption.

Consumption	n	(%)
Less than one glass	37	14.2
One glass	108	41.5
Two glasses	61	23.5
Three glasses	22	8.5
Four and more	12	4.6
doesn’t drink at all	9	3.5
I don’t know/I’m not sure	10	3.8
Missing	1	0.4

**Table A15:** Fruit consumption.

Consumption	n	(%)
One	96	36.9
Two	74	28.5
Three and more	73	28.1
Does not consume fruit	8	3.1
I’m not sure	8	3.1
Missing	1	0.4

**Table A16:** Consumption of vegetables.

Consumption	n	(%)
One	141	54.2
Two	66	25.4
Three and more	37	14.2
Does not consume vegetables	7	2.7
I’m not sure	8	3.1
Missing	1	0.4
